# *PRODH* Polymorphisms, Cortical Volumes and Thickness in Schizophrenia

**DOI:** 10.1371/journal.pone.0087686

**Published:** 2014-02-03

**Authors:** Vanessa K. Ota, Fernanda T. Bellucco, Ary Gadelha, Marcos L. Santoro, Cristiano Noto, Denise M. Christofolini, Idaiane B. Assunção, Karen M. Yamada, Ândrea K. Ribeiro-dos-Santos, Sidney Santos, Jair J. Mari, Marília A. C. Smith, Maria I. Melaragno, Rodrigo A. Bressan, João R. Sato, Andrea P. Jackowski, Sintia I. Belangero

**Affiliations:** 1 Disciplina de Genética, Departamento de Morfologia e Genética, Universidade Federal de Sao Paulo (UNIFESP), Sao Paulo, Sao Paulo, Brazil; 2 LiNC - Laboratório Interdisciplinar de Neurociências Clínicas, Universidade Federal de Sao Paulo (UNIFESP), Sao Paulo, Sao Paulo, Brazil; 3 Departamento de Psiquiatria, Universidade Federal de Sao Paulo (UNIFESP), Sao Paulo, Brazil; 4 Disciplina de Genética e Reproducao Humana, Departamento de Ginecologia e Obstetrícia, Faculdade de Medicina do ABC (FMABC), Santo Andre, Sao Paulo, Brazil; 5 Laboratório de Genética Humana e Médica, Universidade Federal do Pará (UFPA), Belém, Pará, Brazil; 6 Center of Mathematics, Computation and Cognition, Universidade Federal do ABC, Santo Andre, Brazil; The Nathan Kline Institute, United States of America

## Abstract

Schizophrenia is a neurodevelopmental disorder with high heritability. Several lines of evidence indicate that the *PRODH* gene may be related to the disorder. Therefore, our study investigates the effects of 12 polymorphisms of *PRODH* on schizophrenia and its phenotypes. To further evaluate the roles of the associated variants in the disorder, we have conducted magnetic resonance imaging (MRI) scans to assess cortical volumes and thicknesses. A total of 192 patients were evaluated using the Structured Clinical Interview for DSM-IV (SCID), Positive and Negative Syndrome Scale (PANSS), Calgary Depression Scale, Global Assessment of Functioning (GAF) and Clinical Global Impression (CGI) instruments. The study included 179 controls paired by age and gender. The samples were genotyped using the real-time polymerase chain reaction (PCR), restriction fragment length polymorphism (RFLP)-PCR and Sanger sequencing methods. A sample of 138 patients and 34 healthy controls underwent MRI scans. One polymorphism was associated with schizophrenia (rs2904552), with the G-allele more frequent in patients than in controls. This polymorphism is likely functional, as predicted by PolyPhen and SIFT, but it was not associated with brain morphology in our study. In summary, we report a functional *PRODH* variant associated with schizophrenia that may have a neurochemical impact, altering brain function, but is not responsible for the cortical reductions found in the disorder.

## Introduction

Schizophrenia is currently considered to be a neurodevelopmental disease that arises from a complex phase-specific interaction of genetic and environmental factors [1]–[3]. The prevalence of schizophrenia in the general population is estimated to be 0.3%–1.6% [4], [5]. The high heritability (∼64%–83%) of schizophrenia [6], [7] may be due to a combination of multiple common alleles, each one with a small to moderate effect, and some rare alleles with much larger effect sizes [8].

One of the strongest known genetic risk factors for schizophrenia is the 22q11 deletion [9]. Up to one-third of patients with 22q11 deletion syndrome (22q11DS), also known as DiGeorge or velocardiofacial syndrome, develop schizophrenia or schizoaffective disorder [10]. Moreover, while the prevalence of the chromosome 22q11 deletion in the general population is one in 4000 individuals, its frequency is estimated to be approximately 1% in adult patients with schizophrenia [11]–[14]. Therefore, the 22q11DS deletion has been suggested as a genetic subtype of schizophrenia, and the genes located in this region might therefore contribute to susceptibility to the disease.

One of the genes commonly deleted in 22q11DS is the proline dehydrogenase (*PRODH*) gene, which encodes the proline dehydrogenase enzyme that catalyzes the first step in proline catabolism [15]. The *PRODH* gene, consisting of 14 coding exons and located in a low copy repeat sequence, is widely expressed in the brain and in other tissues [16], [17]. The resulting enzyme, also known as proline oxidase (POX), is a mitochondrial tumor suppressor [18] that inhibits proliferation and induces apoptosis [19]. High proline concentrations appear to activate NMDA and AMPA receptors and may therefore act as a neuromodulator [20]–[22]. Proline is also a precursor of the neurotransmitter glutamate [23], which seems to be involved in schizophrenia pathophysiology. Moreover, whereas high levels of plasma proline are found on a relatively common basis in patients with 22q11DS [24], [25], some evidence suggests that hyperprolinemia may lead to neurocognitive dysfunction in animal models [26], [27] and may be involved in the cognitive and psychiatric features of 22q11DS [25].

Patients with 22q11.2 deletion syndrome present abnormalities in brain structure, such as a global brain volumetric reduction that includes several cortical regions, the cerebellum and the hippocampus [28]. Similarly, patients with schizophrenia consistently present brain volumetric reductions compared to healthy controls, specifically due to grey matter reductions of the anterior cingulate, frontal (particularly medial and inferior) and temporal lobes, hippocampus/amygdala, thalamus, and insula that may be magnified over time [29]. A widespread cortical thickness reduction has also been found in the frontal, parietal, temporal and occipital regions of patients with schizophrenia [30].

Only two previous studies have investigated the role of *PRODH* in brain volume. The first, employing a sample of 92 healthy controls, observed a decrease in the striatal grey matter volumes of carriers of the functional risk haplotype, which contains the rs4819756, rs2870983, and rs450046 minor alleles (haplotype: GCC) [31]. The other study investigated two other *PRODH* single nucleotide polymorphisms (SNPs - rs2008720 and rs372055) with respect to brain anatomy in a sample of 51 patients with schizophrenia. While no association was found for rs2008720, significant differences in grey matter (GM) and total brain volume were revealed for rs372055, with C carriers showing increased total GM and total brain volume in comparison with patients carrying the TT genotype [32].

However, the associations of other important functional *PRODH* SNPs with brain morphology have not been investigated, and neither previous study investigated the different roles of these polymorphisms in both normal and pathological conditions. In the present study, we aimed to associate 12 *PRODH* polymorphisms, and especially functional variants, with schizophrenia and other clinical variables such as treatment resistance, age of onset, and positive and negative symptoms. To further understand the impacts of the studied polymorphisms, we conducted MRI scans in a subsample of patients.

## Materials and Methods

### Ethics Statement

This study was approved by the Research Ethics Committee of UNIFESP [CEP No. 1737/06]. Written informed consent was obtained from all recruited participants or their caregivers, and the clinical and laboratory investigations were strictly conducted according to the principles expressed in the Declaration of Helsinki. All invited subjects agreed to enroll in this study. If the patients were within a stable period according to their clinical features and could understand the consent forms, they double-signed the documents themselves. The next of kin, caretakers or guardians consented on the behalf of those participants whose capacity to consent was compromised.

### Subjects

A total of 192 chronically medicated outpatients were recruited from the Schizophrenia Program (PROESQ) at the Universidade Federal de São Paulo (UNIFESP). The diagnosis of schizophrenia was established according to the criteria of the Diagnostic and Statistical Manual of Mental Disorders, Fourth Edition (DSM-IV), using the Structured Clinical Interview of the DSM-IV (SCID). Other clinical variables were assessed using the Positive and Negative Syndrome Scale (PANSS); Calgary Depression Scale [33]; Global Assessment of Functioning (GAF); and Clinical Global Impression (CGI) [34] instruments. All available information, including medical records, were used to assess remission, which was defined by mild severity (score of 3 or lower) in eight items of the PANSS maintained for the 6 months before the assessment, as previously proposed [35]. The illness duration was directly assessed with the patient, and when in doubt, a companion or informant was questioned and/or the information was recovered from the available clinical records. Symptom clusters (Negative, Positive, Disorganization, Excited and Anxiety/Depression) were classified according to the PANSS ratings [35]. Treatment-resistant (TR) status was defined, in accordance with the International Psychopharmacological Criteria (IPAP) [www.ipap.org], as a failure to respond to 4- to 6-week trials of monotherapy with two different antipsychotics at adequate doses (equivalent to 5 mg of risperidone or 400 mg of chlorpromazine).

A 1.5 Mb microdeletion in 22q11.2 was detected in one patient with schizophrenia using multiplex ligation-dependent probe amplification [11]. Therefore, this subject was excluded from the analyses, as this deletion could be a confounding genotype factor.

A total of 179 healthy subjects with no family history of severe psychiatric illness were recruited by UNIFESP. These subjects were also investigated using a modified version of SCID screening to exclude any with a previous illness episode with psychotic features or a family history of similar diseases.

Considering that the Brazilian population is admixed, the population structure was verified for a sample of our study population (Table 1).

**Table 1:** Descriptive characteristics of the study population.

Variables	SCZ patients		Controls		p-values
	N	Frequency/values	N	Frequency/values	
**Gender**	192		179		0.375
Male	131	68.2%	114	63.7%	
Female	61	31.8%	65	36.3%	
**Age (years)**	192	35.68±10.38	178	38.29±12.88	0.033
**Ancestry**					
European	164	0.67±0.22	137	0.69±0.19	0.278
African	164	0.18±0.17	137	0.17±0.16	0.454
Native-American	164	0.15±0.14	137	0.14±0.11	0.426
**Age at onset**	176	23.32±7.24			
**Treatment response**	145				
TR	71	50.3%			
NTR	70	49.7%			
**GAF**	144	50.00±13.29			
**CGI**	144	3.84±1.07			
**PANSS - Negative symptoms**	144	25.24±8.04			
**PANSS – Positive symptoms**	143	16.88±5.98			
**PANSS - Excited**	143	7.31±2.51			
**PANSS – Anxiety/depression**	144	9.21±3.23			
**Calgary**	144	2.79±3.87			

TR: Treatment resistant; NTR: Non-treatment resistant; GAF: Global Assessment of Functioning; CGI: Clinical Global Impression; PANSS: Positive and Negative Syndrome Scale; N: sample size; SCZ: schizophrenia.

### DNA Isolation and Population Structure Analysis

Whole blood was collected into EDTA tubes and genomic DNA isolation was performed using the Gentra Puregene Kit (Qiagen, Maryland, USA) according to the manufacturer’s protocol.

The population genetic ancestry structure was assessed by a panel of 48 ancestry-informative insertion-deletion polymorphisms, previously described and validated in the Brazilian population [36], analyzed in three multiplex PCR assays followed by capillary electrophoresis.

### *PRODH* Genotyping

**Figure 1:**
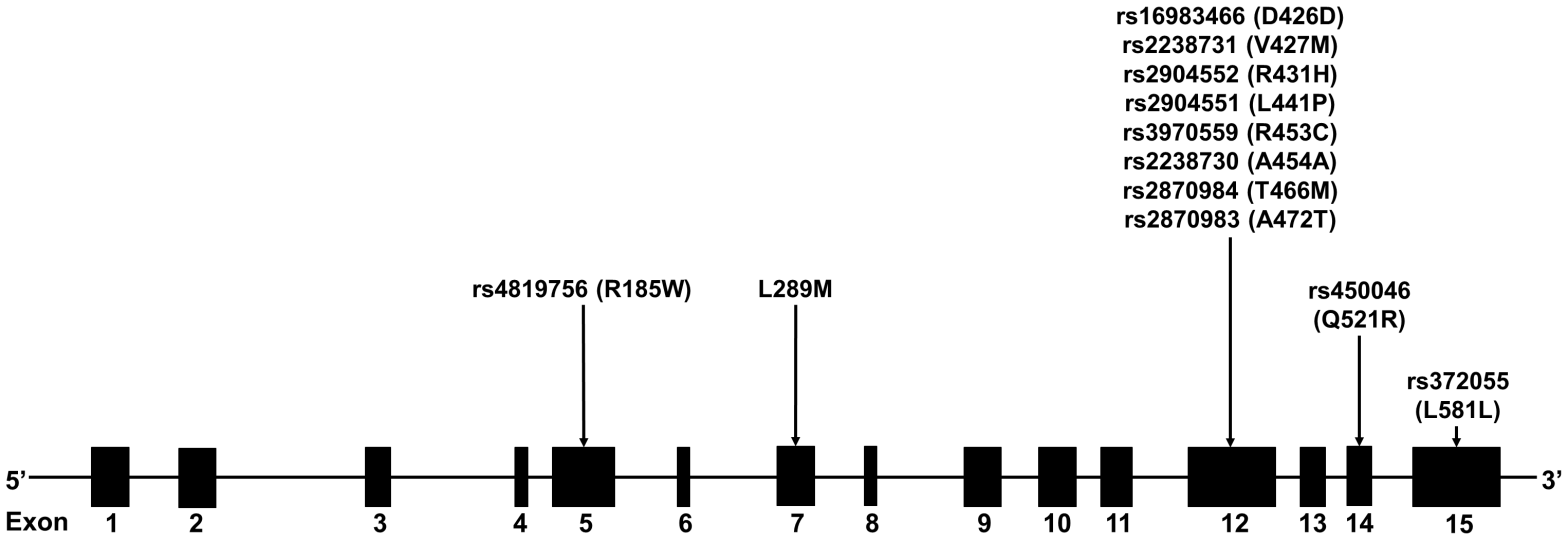
Diagram of the *PRODH* gene, representing the variants investigated in this study and their locations.

A total of 12 *PRODH* SNPs were interrogated (Figure 1) by real-time polymerase chain reaction (PCR), restriction fragment length polymorphism (RFLP)-PCR and sequencing techniques. For real-time PCR, three SNPs (rs4819756 in exon 5, rs450046 in exon 14 and rs372055 in exon 15) were genotyped using commercially available predesigned TaqMan® probes and primers (Applied Biosystems, Foster City, USA), as described by the manufacturer. One polymorphism (L289M in exon 8) was investigated by the RFLP-PCR technique using the *BsaBI* enzyme and the following PCR primers: 5′-TGGTGGGGAGGAGGAGGTCA-3′ (forward) and 5′-CAGCCAGGACTGGGAGACGT-3′ (reverse). The other eight SNPs in exon 12 (rs16983466, rs2238731, rs2904552, rs2904551, rs3970559, rs2238730, rs2870984 and rs2870983) were investigated using Sanger sequencing. The PCR primers were as follow: 5’-TCCCCACTGCCATTGCTCCT-3′ (forward) and 5′-CCTGCCCTGAGAAGACAGAG-3′ (reverse). The PCR conditions are available upon request.

### Genetic Analysis

The characteristics of the study population are described in Table 1. First, the Hardy-Weinberg equilibrium was verified for all SNPs using the chi-square test. Logistic regression was then performed to associate each genotype with the diagnosis of schizophrenia, using age as a covariate. The Bonferroni correction for multiple comparisons was performed. The linkage disequilibrium and haplotypes were assessed using Haploview [37]. The associations between the haplotypes and the disease were investigated using the chi-square test.

Statistical analyses were performed using the Statistical Package for the Social Sciences (SPSS version 15.0), and the level of significance was set at 0.05.

### *In silico* Analysis

For the SNPs associated with schizophrenia, we tested the predictive values using SIFT (http://sift.jcvi.org/) and PolyPhen (http://genetics.bwh.harvard.edu/pph/) and analyzed the amino acid conservation using the UCSC genome browser (http://genome.ucsc.edu). Both SIFT and PolyPhen predict whether an amino acid substitution affects protein function. SIFT prediction is based on the degree of conservation of amino acid residues in sequence alignments [38], while PolyPhen uses straightforward physical and comparative considerations [39].

### Imaging Acquisition

Brain MRIs were obtained from 138 subjects with schizophrenia (93 men, age  = 35.21±10.21; 45 women, age  = 39.27±11.24) and 34 healthy controls (23 men, age  = 32.43±7.33; 11 women, age  = 38.73±15.78) using a 1.5T scanner [Magnetom Sonata (Maestro Class) Siemens AG, Medical Solutions, Erlangen, Germany] with an eight-channel head coil. A series of exploratory sagittal images (nine to eleven slices of 5 mm with 1 mm spacing) was performed to evaluate the image quality and the positioning of the head of each subject. T1 images were acquired sequentially using a pulse sequence (SPGR) with the following parameters: TR  = 2000 ms, TE  = 3.42 ms, matrix size  = 256 x 256, FOV  = 245 mm, flip angle  = 15 degrees, NEX  = 1, 1.0 mm slice thickness with no gaps, yielding 192 slices.

### Imaging Preprocessing – FreeSurfer

The MRI scans were analyzed using the FreeSurfer software package (version 5.0 http://surfer.nmr.mgh.harvard.edu) based on established processing steps (recon-all pipeline). The cortical volumes and thickness information of the brain regions were estimated using the automated parcellation of the Desikan-Killiany Atlas [40] and exported to SPSS for further analysis.

### Imaging Group Analysis

First, a general linear model (GLM) was used to assess the association of each cortical and subcortical volume and thickness with schizophrenia. Then, all measures were inserted as dependent variables, and each SNP, schizophrenia diagnosis and the interaction between them were included as fixed factors. The total intracranial volume (TIV), gender and age were included as covariates. Statistical significance was set *a priori* at a two-tailed alpha of 0.05.

## Results

### Association between *PRODH* Variants and Schizophrenia

The genotype and allele frequencies for each variant are represented in Table S1. The genotype distributions did not deviate from those predicted by the Hardy-Weinberg equilibrium in both the case and control groups (p>0.05) for all variants, except for rs4819756 (controls: χ^2^ = 4.38, df  = 1, p  = 0.036; cases: χ^2^ = 9.70, df  = 1, p  = 0.002) and rs450046 (cases: χ^2^ = 4.20, df  = 1, p  = 0.040).

Regarding allele comparisons, one polymorphism was associated with schizophrenia [rs2904552 (G-allele): p  = 0.002, OR  = 2.65, 95%CI  = 1.45–4.86] after Bonferroni correction (adjusted p-value  = 0.024). Comparing the genotypes of rs2904552, we found that the GG-genotype carriers were at greater risk for a schizophrenia diagnosis in comparison to the AG-genotype carriers (adjusted p-value  = 0.048, OR  = 2.61, 95%CI  = 1.36–5.03). Comparing the GG homozygotes to the A-allele carriers (grouping GA and AA genotype carriers), a stronger association was found (adjusted p-values  = 0.024; OR  = 2.77; 95%CI  = 1.45–5.30). In all these analyses, the power was >80% as determined using G*Power 3.1.6.

For the haplotype analyses, only polymorphisms in Hardy-Weinberg equilibrium and with a minor allele frequency >5% were included (rs16983466, rs2238731, rs2904552, rs2870983 and rs372055). The results of the linkage disequilibrium test are described in Figure S1. One haplotype block composed of three variants, and five composed of two variants, were analyzed (Table S2). Concerning the haplotype composed by three variants, a significant association was detected between the A/G/C haplotype (rs2904552/rs2238731/rs16983466) and schizophrenia (p  = 0.001). Analyzing the combinations of two variants, only those haplotypes constructed with rs2904552 SNP remained significant (p<0.05– Table S2).

### Association between *PRODH* Variants and Schizophrenia Phenotypes

We analyzed the association of age at onset, TR, GAF, CGI, Calgary, Negative, Positive, Disorganized, Excited and Anxiety/Depression clusters with each *PRODH* polymorphism, but no significant associations were detected.

### *In silico* Analysis

To predict the likely pathogenicity of rs2904552, we conducted an *in silico* analysis. This SNP seems to have a functional role, as it was classified as “probably damaging,” with a score of 0.996, using PolyPhen and as “damaging,” with a score of 0.04, using SIFT. Data were also available for five orthologous species (three mammals, one frog and one fish), and the residues showed evolutionary conservation in all these organisms. Furthermore, this variation was reported in a previous study to decrease the enzyme activity [41].

### Association between MRI and Schizophrenia

For the cortical and subcortical volumes, 21 regions were associated with schizophrenia after Bonferroni correction and are described in Table S3. In terms of cortical thickness, seven regions differed between the cases and controls (Table S4).

### Association between *PRODH* Variants and MRI

In the comparison of all *PRODH* genotypes with all brain volumes and cortical thicknesses, only one association remained significant after Bonferroni correction: the interaction between rs2238731 (V427M) and schizophrenia was associated with right pars opercularis volume (p  = 0.00020; effect size  = 0.081; power  = 0.965). Controls carrying the GA genotype had an increased volume (6261.50±144.95 mm^3^) in this region compared to carriers of the GG genotype (4193.37±628.09 mm^3^). However, in schizophrenia patients, GA genotype carriers had a smaller volume (3796.68±527.12 mm^3^) than did GG carriers (3993.41±733.26 mm^3^).

## Discussion

In this study, one *PRODH* variant (rs2904552 or R431H) and its haplotypes were associated with schizophrenia, with the GG genotype present at a higher frequency among patients than among healthy controls. To further understand the role of this SNP, we investigated its role in brain volumes and cortical thicknesses.

### *PRODH* Polymorphisms and Schizophrenia

Among the 12 investigated SNPs, 10 had been previously associated with schizophrenia (rs372055, rs450046), hyperprolinemia (L289M, rs2870983, rs4819756, rs2904552, rs2870984) or both (rs2904551, rs2238731, rs3970559); two SNPs had not been previously investigated (rs16983466, rs2238730).

Different lines of evidence suggest an important role for *PRODH* in schizophrenia: 1) it is located at 22q11.2, the deletion of which is one of the strongest genetic risk factors for schizophrenia; 2) *PRODH* deletion missense mutations have been described in patients with schizophrenia [41], [42]; 3) an association between schizoaffective disorders and moderate hyperprolinemia was previously been detected [43] *;* 4) it is widely expressed in the brain; 5) mice with PRODH deficiency show elevated plasma and brain proline levels, locally decreased glutamate and γ-aminobutyric acid (GABA) levels and a deficit in sensorimotor gating [17], [44]; 6) the PRODH enzyme is rate limiting in the conversion of proline to glutamate [41], which is important for signal transduction between neurons and for synaptic plasticity [45] and seems to be deregulated in schizophrenia [46].

Indeed, a recent metabolomics study reported abnormalities related to proline metabolism in schizophrenia [47]. Furthermore, Savio et al. (2012) reported that long-term exposure to proline induced behavioral changes in adult zebrafish that were reversed by the acute administration of atypical antipsychotics [48]. In this vein, several studies have investigated peripheral proline levels as a risk factor for schizophrenia (reporting no association) [49], [50] or the positive associations between hyperprolinemia and schizoaffective disorder [43] and schizophrenia in women [51]. Despite these mixed findings, the data still support a functional role for *PRODH* variants and hyperprolinemia in the pathophysiology of schizophrenia [31].

Along with its role in proline catabolism, PRODH is also known as a key enzyme in controlling homeostasis, providing energy and shuttling redox potential between cellular compartments and the production of reactive oxygen species [52].

*PRODH* rs2904552 is a nonsynonymous mutation that causes the substitution of an arginine for a histidine in codon 431. This change seems to promote a functional effect, according to our *in silico* findings, and also results in a moderate decrease (30–70%) of PRODH enzyme activity [41]. Also, another study has detected this polymorphism in combination with L441P in a subject with high levels of proline [42]. Two other studies have investigated this mutation; however, neither of them reported a positive association with schizophrenia [43], [53]. As we detected an association between the major G-allele and schizophrenia, we suggest that the minor A-allele might be a protective factor and may thus prevent the onset of the disease by altering the enzyme activity in brain.

### MRI

Our results support earlier findings of reduced total, left and right hemisphere gray matter volumes, corpora callosa, frontal, parietal and temporal lobes, and hippocampi, all of which are brain regions involved in the pathophysiology of schizophrenia [29]. We also found a significant association between the interaction of rs2238731 and schizophrenia diagnosis with the right pars opercularis. Controls carrying the GA genotype had an increased volume in this region compared to GG genotype carriers; however, patients carrying the GA genotype had a smaller volume than did GG patients. This result should be further verified in a larger sample due to the small sample of the GA genotype carriers in the both the control (*N  = *2) and patient (*N  = *19) groups.

### Limitations

Some limitations of this study should be noted. First, we should have analyzed plasma proline levels. Considering that rs2904552 variant may be affecting PRODH enzyme, its substrate might be dysregulated. For this measurement, gender, acute alcohol use and medication, such as valproic acid, may be taken into consideration [43], [54], [55]. Furthermore, the duration of illness and medications may have been confounding factors for the neuroimaging analysis.

## Conclusions

This study represents the first report of the effects of *PRODH* polymorphisms on brain morphology in both patients and healthy controls, testing for the interaction between this SNP and a diagnosis of schizophrenia. Only one SNP (rs2238731) seemed to be significantly associated with right pas opercularis volume, but this finding should be confirmed in larger samples. Therefore, considering the functional effect of rs2904552 and the importance of proline levels in schizophrenia, this SNP may act by altering neurochemistry and is not responsible for the deficits in brain structure found in schizophrenia.

In summary, this study indicates that the *PRODH* rs2904552 polymorphism may be a genetic risk factor for developing schizophrenia, and further studies should address how disturbance of the proline pathway could affect brain function.

## Supporting Information

Figure S1 **Linkage disequilibrium plot across the proline dehydrogenase (** ***PRODH*** **) gene. Numbers within the diamonds are D' values for the respective SNP pairs.**
(TIF)

Table S1 **Genotype and allele frequencies of** ***PRODH*** **variants.**
(DOCX)

Table S2 **Haplotype frequencies of PRODH variants in patient and control group.**
(DOCX)

Table S3 **Association between cortical and subcortical volumes and schizophrenia.**
(DOCX)

Table S4 **Association between cortical thickness and schizophrenia.**
(DOCX)
