# Supplementary figures and images for: PRODH Polymorphisms, Cortical Volumes and Thickness in Schizophrenia
Source: PLoS One. 2014 Feb 3;9(2):e87686. doi: 10.1371/journal.pone.0087686 (PMC3912045; doi:10.1371/journal.pone.0087686)

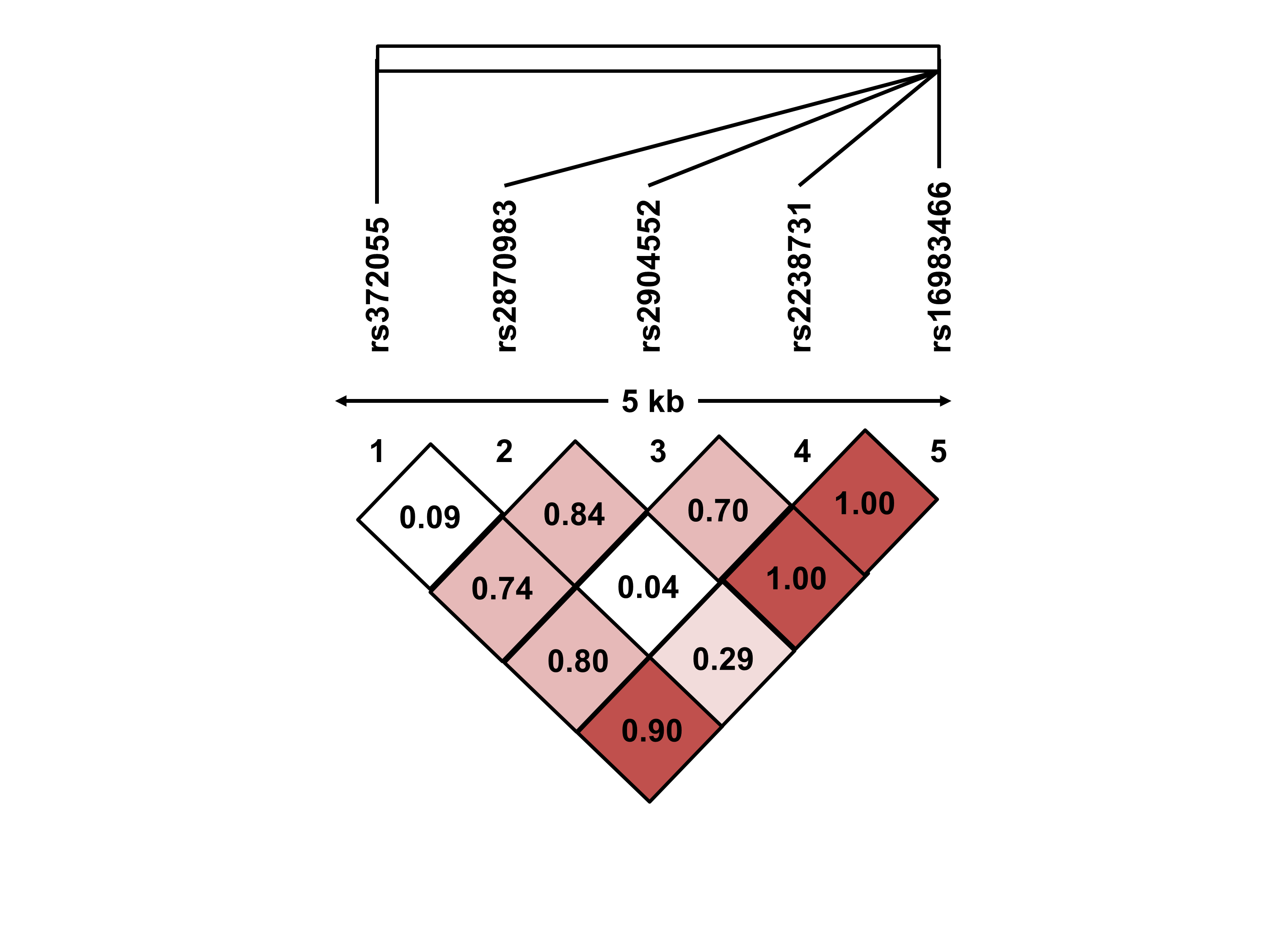

Supplement: Figure S1 — Linkage disequilibrium plot across the proline dehydrogenase ( PRODH ) gene. Numbers within the diamonds are D' values for the respective SNP pairs. (TIF) [file pone.0087686.s001.tif]
