# Supplementary material for: PRODH Polymorphisms, Cortical Volumes and Thickness in Schizophrenia
Source: PLoS One. 2014 Feb 3;9(2):e87686. doi: 10.1371/journal.pone.0087686 (PMC3912045; doi:10.1371/journal.pone.0087686)
Supplement: Table S1 — Genotype and allele frequencies of PRODH variants. (DOCX) [file pone.0087686.s002.docx]

**Table S1.** Genotype and allele frequencies of *PRODH* variants.

| **Group** | **N** | **Genotype (%)** | | | **Allele** | |
| --- | --- | --- | --- | --- | --- | --- |
| **rs4819756 (R185W)** |  | **GG** | **GA** | **AA** | **G** | **A** |
| Patients | 184 | 108 (58.7) | 55 (29.9) | 11 (11.4) | 77.87% | 22.13% |
| Controls | 175 | 83 (47.4) | 68 (38.9) | 24 (13.7) | 66.86% | 33.14% |
| **L289M** |  | **TT** | **TA** | **AA** | **T** | **A** |
| Patients | 191 | 188 (98.4) | 3 (1.6) | 0 (0.0) | 99.21% | 0.79% |
| Controls | 165 | 163 (98.8) | 2 (1.2) | 0 (0.0) | 99.39% | 0.61% |
| **rs16983466 (D426D)** |  | **CC** | **CT** | **TT** | **C** | **T** |
| Patients | 191 | 126 (66.0) | 61 (31.9) | 4 (2.1) | 81.94% | 18.06% |
| Controls | 179 | 118 (65.9) | 52 (29.1) | 9 (5.0) | 80.45% | 19.55% |
| **rs2238731 (V427M)** |  | **GG** | **GA** | **AA** | **G** | **A** |
| Patients | 192 | 166 (86.5) | 26 (13.5) | 0 (0) | 93.23% | 6.77% |
| Controls | 179 | 165 (92.2) | 14 (7.8) | 0 (0) | 96.09% | 3.91% |
| **rs2904552 (R431H)** |  | **GG** | **GA** | **AA** | **G** | **A** |
| Patients | 192 | 176 (91.7) | 16 (8.3) | 0 (0) | 95.83% | 4.17% |
| Controls | 179 | 144 (80.4) | 33 (18.4) | 2 (1.1) | 89.66% | 10.34% |
| **rs2904551 (L441P)** |  | **TT** | **TC** | **CC** | **T** | **C** |
| Patients | 192 | 189 (98.4) | 3 (1.6) | 0 (0) | 99.22% | 0.78% |
| Controls | 179 | 173 (96.6) | 6 (3.4) | 0 (0) | 98.32% | 1.68% |
| **rs3970559 (R453C)** |  | **CC** | **CT** | **TT** | **C** | **T** |
| Patients | 192 | 187 (97.4) | 5 (2.6) | 0 (0) | 98.70% | 1.30% |
| Controls | 179 | 170 (95.0) | 9 (5.0) | 0 (0) | 97.49% | 2.51% |
| **rs2238730 (A454A)** |  | **GG** | **GA** | **AA** | **G** | **A** |
| Patients | 192 | 183 (95.3) | 9 (4.7) | 0 (0) | 97.66% | 2.34% |
| Controls | 179 | 172 (96.1) | 7 (3.9) | 0 (0) | 98.04% | 1.96% |
| **rs2870984 (T466M)** |  | **CC** | **CT** | **TT** | **C** | **T** |
| Patients | 192 | 184 (95.8) | 8 (4.2) | 0 (0) | 97.92% | 2.08% |
| Controls | 179 | 174 (97.2) | 5 (2.8) | 0 (0) | 98.60% | 1.40% |
| **rs2870983 (A472T)** |  | **GG** | **GA** | **AA** | **G** | **A** |
| Patients | 192 | 159 (82.8) | 31 (16.1) | 2 (1.1) | 90.89% | 9.11% |
| Controls | 179 | 160 (89.4) | 19 (10.6) | 0 (0) | 94.69% | 5.31% |
| **rs4550046 (Q521R)** |  | **TT** | **TC** | **CC** | **T** | **C** |
| Patients | 191 | 175 (91.6) | 15 (7.9) | 1 (0.5) | 95.55% | 4.45% |
| Controls | 177 | 158 (89.3) | 19 (10.7) | 0 (0.0) | 94.63% | 5.37% |
| **rs372055 (L581L)** |  | **AA** | **AG** | **GG** | **A** | **G** |
| Patients | 191 | 109 (57.1) | 67 (35.1) | 15 (7.8) | 74.61% | 25.39% |
| Controls | 179 | 88 (49.2) | 80 (44.7) | 11 (6.1) | 71.51% | 28.49% |

N: Sample size
