# Supplementary material for: PRODH Polymorphisms, Cortical Volumes and Thickness in Schizophrenia
Source: PLoS One. 2014 Feb 3;9(2):e87686. doi: 10.1371/journal.pone.0087686 (PMC3912045; doi:10.1371/journal.pone.0087686)
Supplement: Table S2 — Haplotype frequencies of PRODH variants in patient and control group. (DOCX) [file pone.0087686.s003.docx]

**Table S2.** Haplotype frequencies of *PRODH* variants in patient and control group.

| **Haplotype** | **Total** | **Patients** | **Controls** | **χ^2^** | **p-value** |
| --- | --- | --- | --- | --- | --- |
| **rs2904552/rs2238731/rs16983466** |  |  |  |  |  |
| G/G/C | 0.687 | 0.710 | 0.662 | 2.016 | 0.156 |
| G/G/T | 0.188 | 0.180 | 0.196 | 0.284 | 0.594 |
| A/G/C | 0.071 | 0.042 | 0.103 | 10.269 | 0.001* |
| G/A/C | 0.054 | 0.068 | 0.039 | 2.972 | 0.085 |
| **rs2904552/rs2238731** |  |  |  |  |  |
| G/G | 0.876 | 0.891 | 0.859 | 1.725 | 0.189 |
| A/G | 0.070 | 0.041 | 0.102 | 10.356 | 0.001* |
| G/A | 0.053 | 0.067 | 0.037 | 3.308 | 0.069 |
| **rs2238731/rs16983466** |  |  |  |  |  |
| G/C | 0.758 | 0.752 | 0.765 | 0.192 | 0.661 |
| G/T | 0.188 | 0.181 | 0.196 | 0.266 | 0.606 |
| A/C | 0.054 | 0.068 | 0.039 | 2.972 | 0.085 |
| **rs2904552/rs16983466** |  |  |  |  |  |
| G/C | 0.741 | 0.778 | 0.701 | 5.724 | 0.017* |
| G/T | 0.188 | 0.180 | 0.196 | 0.285 | 0.593 |
| A/C | 0.071 | 0.042 | 0.103 | 10.629 | 0.001 |
| **rs2870983/rs2904552** |  |  |  |  |  |
| G/G | 0.856 | 0.867 | 0.844 | 0.837 | 0.360 |
| A/G | 0.073 | 0.091 | 0.053 | 3.98 | 0.046* |
| G/A | 0.071 | 0.042 | 0.103 | 10.629 | 0.001* |
| **rs372055/rs16983466** |  |  |  |  |  |
| A/C | 0.718 | 0.730 | 0.705 | 0.579 | 0.447 |
| G/T | 0.174 | 0.163 | 0.186 | 0.677 | 0.410 |
| G/C | 0.094 | 0.090 | 0.099 | 0.192 | 0.662 |
| A/T | 0.013 | 0.017 | 0.010 | 0.717 | 0.397 |

N: Sample size
