# Supplementary material for: PRODH Polymorphisms, Cortical Volumes and Thickness in Schizophrenia
Source: PLoS One. 2014 Feb 3;9(2):e87686. doi: 10.1371/journal.pone.0087686 (PMC3912045; doi:10.1371/journal.pone.0087686)
Supplement: Table S3 — Association between cortical and subcortical volumes and schizophrenia. (DOCX) [file pone.0087686.s004.docx]

**Table S3.** Association between cortical and subcortical volumes and schizophrenia.

| **Brain region** | **Controls Mean Volume ± SD (mm^3^)** | **Patients Mean Volume ± SD (mm^3^)** | **p-value** | **Partial Eta squared** | **Power** |
| --- | --- | --- | --- | --- | --- |
| Left Cerebellum White Matter | 14310.91±2094.69 | 14527.51±1789.30 | 0.706 | 0.001 | 0.066 |
| Left Cerebellum Cortex | 56643.79±5888.25 | 57156.27±6547.94 | 0.928 | 0.000 | 0.051 |
| Left Thalamus Proper | 7855.38±802.72 | 7295.92±978.31 | <0.001** | 0.115 | 0.996 |
| Left Caudate | 3737.53±543.75 | 3719.01±552.54 | 0.593 | 0.002 | 0.083 |
| Left Putamen | 5608.29±599.17 | 5645.45±805.68 | 0.980 | 0.000 | 0.050 |
| Left Pallidum | 1765.50±266.03 | 1843.49±268.85 | 0.129 | 0.014 | 0.329 |
| Brain Stem | 22855.29±2524.99 | 22339.17±2655.39 | 0.034* | 0.027 | 0.568 |
| Left Hippocampus | 4339.68±365.20 | 4087.31±492.96 | <0.001** | 0.087 | 0.976 |
| Left Amygdala | 1553.06±132.23 | 1533.13±211.62 | 0.231 | 0.009 | 0.223 |
| Left Accumbens area | 543.12±118.52 | 525.20±102.90 | 0.165 | 0.012 | 0.284 |
| Right Cerebellum White Matter | 13976.89±1800.16 | 14050.31±1701.28 | 0.903 | 0.000 | 0.052 |
| Right Cerebellum Cortex | 58210.15±6458.08 | 58700.77±6584.45 | 0.931 | 0.000 | 0.051 |
| Right Thalamus Proper | 7831.00±800.02 | 7373.28±934.14 | <0.001** | 0.114 | 0.996 |
| Right Caudate | 3652.56±607.33 | 3584.75±508.27 | 0.250 | 0.008 | 0.209 |
| Right Putamen | 5382.59±598.42 | 5408.53±766.49 | 0.913 | 0.000 | 0.051 |
| Right Pallidum | 1591.35±200.45 | 1639.76±230.54 | 0.293 | 0.007 | 0.183 |
| Right Hippocampus | 4341.91±377.04 | 4157.91±492.62 | 0.001* | 0.061 | 0.905 |
| Right Amygdala | 1630.97±169.72 | 1572.08±211.53 | 0.016* | 0.035 | 0.679 |
| Right Accumbens area | 513.68±80.82 | 512.72±101.16 | 0.723 | 0.001 | 0.064 |
| CC Posterior | 881.97±140.25 | 864.95±153.30 | 0.327 | 0.006 | 0.165 |
| CC Mid Posterior | 457.73±94.58 | 417.94±90.70 | 0.016* | 0.034 | 0.677 |
| CC Central | 489.47±111.15 | 438.02±98.40 | 0.008* | 0.042 | 0.765 |
| CC Mid Anterior | 510.64±137.41 | 449.72±100.89 | 0.003* | 0.053 | 0.858 |
| CC Anterior | 881.21±146.62 | 846.88±158.36 | 0.150 | 0.012 | 0.301 |
| Left Cortex Volume | 238741.28±20276.58 | 225104.74±24697.16 | <0.001** | 0.159 | 10.000 |
| Right Cortex Volume | 241092.88±19174.10 | 226631.17±24615.64 | <0.001** | 0.184 | 10.000 |
| Cortex Volume | 479834.17±39237.42 | 451735.92±49194.35 | <0.001** | 0.173 | 10.000 |
| Left Caudal anterior-cingulate | 2015.73±565.78 | 1760.23±514.16 | 0.003* | 0.051 | 0.841 |
| Left Caudal middle frontal | 6853.29±1124.10 | 6328±1257.28 | 0.002* | 0.056 | 0.880 |
| Left Cuneus | 2778.79±534.71 | 2732.93±482.82 | 0.290 | 0.007 | 0.184 |
| Left Entorhinal | 2049.00±352.47 | 1978.40±440.77 | 0.147 | 0.013 | 0.305 |
| Left Fusiform | 10129.56±1488.79 | 9692.48±1478.50 | 0.039* | 0.025 | 0.542 |
| Left Inferior parietal | 12977.05±1928.55 | 12107.82±1702.11 | 0.001* | 0.061 | 0.905 |
| Left Inferior temporal | 11800.82±2064.51 | 11169.34±1971.10 | 0.013* | 0.037 | 0.706 |
| Left Isthmus– cingulate | 2735.97±526.81 | 2585.34±460.91 | 0.010* | 0.039 | 0.738 |
| Left Lateral occipital | 11810.32±1509.91 | 11355.16±1534.48 | 0.015* | 0.035 | 0.684 |
| Left Lateral orbitofrontal | 8500.35±848.76 | 7947.93±1069.24 | <0.001** | 0.093 | 0.984 |
| Left Lingual | 6340.76±1063.93 | 6098.27±1032.80 | 0.083 | 0.018 | 0.410 |
| Left Medial orbital frontal | 6211.58±893.10 | 5727.16±853.13 | <0.001** | 0.087 | 0.976 |
| Left Middle temporal | 11156.44±1901.17 | 10707.02±1748.82 | 0.019* | 0.032 | 0.651 |
| Left Parahippocampal | 2262.17±321.78 | 2113.45±349.07 | 0.008* | 0.042 | 0.761 |
| Left Paracentral | 3305.70±477.20 | 3091.49±544.31 | 0.002* | 0.058 | 0.890 |
| Left Pars opercularis | 5088.06±816.29 | 4916.52±948.61 | 0.226 | 0.009 | 0.227 |
| Left Pars orbitalis | 2391.85±382.20 | 2316.25±353.87 | 0.142 | 0.013 | 0.311 |
| Left Pars triangularis | 4054.11±683.16 | 3693.39±689.65 | 0.003* | 0.050 | 0.838 |
| Left Pericalcarine | 1940.73±351.82 | 1802.67±346.83 | 0.002* | 0.054 | 0.865 |
| Left Postcentral | 9270.08±916.06 | 8774.37±1275.19 | 0.001* | 0.064 | 0.916 |
| Left Posterior-cingulate | 3326.58±633.95 | 3050.44±511.27 | <0.001** | 0.079 | 0.964 |
| Left Precentral | 12467.52±1231.91 | 11864.44±1481.35 | 0.001* | 0.067 | 0.929 |
| Left Precuneus | 9356.00±1211.18 | 8865.86±1256.84 | 0.002* | 0.058 | 0.890 |
| Left Rostral anterior cingulate | 2969.26±682.70 | 2768.98±538.93 | 0.010* | 0.040 | 0.741 |
| Left Rostral middle frontal | 17398.29±2175.59 | 15881.09±2388.44 | <0.001** | 0.128 | 0.998 |
| Left Superior frontal | 23467.64±2475.50 | 21567.42±2681.90 | <0.001** | 0.146 | 10.000 |
| Left Superior parietal | 12632.82±1437.93 | 12218.57±1898.39 | 0.032* | 0.027 | 0.574 |
| Left Superior Temporal | 12494.94±1387.49 | 11710.65±1637.12 | <0.001** | 0.074 | 0.951 |
| Left Supramarginal | 11092.70±1315.57 | 10376.11±1552.92 | <0.001** | 0.082 | 0.969 |
| Left Frontal pole | 850.58±176.20 | 784.33±151.70 | 0.025* | 0.030 | 0.614 |
| Left Temporal pole | 2407.68±325.79 | 2499.82±505.67 | 0.415 | 0.004 | 0.128 |
| Left Transverse temporal | 1166.61±185.33 | 1090.33±230.76 | 0.031* | 0.028 | 0.582 |
| Left Insula | 7463.85±913.05 | 7113.06±940.79 | 0.001* | 0.063 | 0.912 |
| Right Caudal anterior-cingulate | 2273.67±509.31 | 2095.16±496.18 | 0.017* | 0.034 | 0.672 |
| Right Caudal middle frontal | 6097.62±1111.03 | 5866.39±1176.81 | 0.126 | 0.014 | 0.334 |
| Right Cuneus | 3153.56±422.78 | 3040.72±567.50 | 0.075 | 0.019 | 0.429 |
| Right Entorhinal | 1866.00±403.64 | 1842.21±433.30 | 0.369 | 0.005 | 0.146 |
| Right Fusiform | 10172.11±1485.72 | 9320.10±1300.16 | <0.001** | 0.097 | 0.987 |
| Right Inferior parietal | 15090.35±1769.82 | 14452.31±1960.18 | 0.017* | 0.034 | 0.671 |
| Right Inferior temporal | 11197.03±1747.42 | 10775.81±1794.70 | 0.055 | 0.022 | 0.485 |
| Right Isthmus– cingulate | 2523.29±443.23 | 2363.48±387.82 | 0.002* | 0.054 | 0.867 |
| Right Lateral occipital | 11971.91±1839.32 | 11391.31±1737.69 | 0.003* | 0.053 | 0.859 |
| Right Lateral orbitofrontal | 8474.70±1115.08 | 7882.18±1079.03 | <0.001** | 0.098 | 0.988 |
| Right Lingual | 6695.35±1022.24 | 6274.27±1053.27 | 0.004* | 0.048 | 0.822 |
| Right Medial orbital frontal | 5888.23±700.09 | 5348.71±692.18 | <0.001** | 0.148 | 10.000 |
| Right Middle temporal | 12370.26±1577.13 | 11949.52±1930.68 | 0.027* | 0.029 | 0.601 |
| Right Parahippocampal | 2076±305.32 | 1928.28±300.63 | 0.003* | 0.053 | 0.858 |
| Right Paracentral | 3772.94±710.44 | 3508.51±562.87 | 0.002* | 0.057 | 0.884 |
| Right Pars opercularis | 4315.02±784.34 | 3966.12±709.92 | 0.001* | 0.060 | 0.897 |
| Right Pars orbitalis | 3085.20±476.93 | 2847.11±466.21 | 0.001* | 0.070 | 0.938 |
| Right Pars triangularis | 4926.91±671.20 | 4448.69±904.99 | 0.001* | 0.063 | 0.915 |
| Right Pericalcarine | 2340.08±450.88 | 2132.83±436.85 | 0.001* | 0.061 | 0.906 |
| Right Postcentral | 8982.14±925.68 | 8481.76±1238.13 | 0.001* | 0.068 | 0.934 |
| Right Posterior Cingulate | 3268.14±389.15 | 3094.63±544.15 | 0.007* | 0.043 | 0.776 |
| Right Precentral | 12586.08±1692.38 | 12024.83±1639.64 | 0.006* | 0.045 | 0.790 |
| Right Precuneus | 9841.94±1130.81 | 9156.67±1255.50 | <0.001** | 0.107 | 0.993 |
| Right Rostral anterior cingulate | 2451.85±502.70 | 2281.10±485.63 | 0.008* | 0.042 | 0.762 |
| Right Rostral middle frontal | 18189.67±2482.45 | 16693.48±2431.48 | <0.001** | 0.118 | 0.997 |
| Right Superior frontal | 23168.64±2718.79 | 20988.61±2745.37 | <0.001** | 0.175 | 10.000 |
| Right Superior parietal | 12479.06±1679.19 | 12079.38±1704.20 | 0.047* | 0.024 | 0.512 |
| Right Superior Temporal | 12231.91±1340.91 | 11440.53±1603.87 | <0.001** | 0.076 | 0.957 |
| Right Supramarginal | 10442.29±1261.96 | 9792.21±1434.16 | 0.001* | 0.065 | 0.922 |
| Right Frontal pole | 1130.73±242.31 | 1044.22±182.95 | 0.024* | 0.030 | 0.621 |
| Right Temporal pole | 2273.47±503.47 | 2319.71±454.85 | 0.813 | 0.000 | 0.056 |
| Right Transverse temporal | 912.03±133.79 | 857.29±187.43 | 0.077 | 0.019 | 0.423 |
| Right Insula | 7791.73±680.05 | 7329.67±1070.34 | <0.001** | 0.079 | 0.964 |

*p<0.05; **p<0.00051 (Bonferroni correction)
