# Supplementary material for: PRODH Polymorphisms, Cortical Volumes and Thickness in Schizophrenia
Source: PLoS One. 2014 Feb 3;9(2):e87686. doi: 10.1371/journal.pone.0087686 (PMC3912045; doi:10.1371/journal.pone.0087686)
Supplement: Table S4 — Association between cortical thickness and schizophrenia. (DOCX) [file pone.0087686.s005.docx]

**Table S4.** Association between cortical thickness and schizophrenia.

| **Brain region** | **Controls Mean Thickness ± SD (mm)** | **Patients Mean Thickness ± SD (mm)** | **p-value** | **Partial Eta squared** | **Power** |
| --- | --- | --- | --- | --- | --- |
| Left Caudal anterior-cingulate | 2.74±0.33 | 2.67±0.33 | 0.345 | 0.005 | 0.156 |
| Left Caudal middle frontal | 2.64±0.11 | 2.52±0.16 | <0.001** | 0.087 | 0.976 |
| Left Cuneus | 1.80±0.15 | 1.76±0.13 | 0.089 | 0.017 | 0.398 |
| Left Entorhinal | 3.32±0.33 | 3.26±0.37 | 0.403 | 0.004 | 0.132 |
| Left Fusiform | 2.68±0.14 | 2.62±0.15 | 0.015* | 0.035 | 0.682 |
| Left Inferior parietal | 2.55±0.15 | 2.51±0.15 | 0.204 | 0.010 | 0.245 |
| Left Inferior temporal | 2.91±0.15 | 2.87±0.18 | 0.250 | 0.008 | 0.210 |
| Left Isthmus– cingulate | 2.54±0.18 | 2.50±0.22 | 0.474 | 0.003 | 0.110 |
| Left Lateral occipital | 2.22±0.13 | 2.19±0.13 | 0.151 | 0.012 | 0.300 |
| Left Lateral orbitofrontal | 2.85±0.19 | 2.70±0.17 | <0.001** | 0.113 | 0.995 |
| Left Lingual | 1.95±0.14 | 1.90±0.12 | 0.052 | 0.023 | 0.496 |
| Left Medial orbital frontal | 2.73±0.22 | 2.61±0.17 | 0.002* | 0.057 | 0.885 |
| Left Middle temporal | 2.94±0.15 | 2.86±0.18 | 0.038* | 0.026 | 0.548 |
| Left Parahippocampal | 2.74±0.32 | 2.60±0.29 | 0.014* | 0.036 | 0.692 |
| Left Paracentral | 2.25±0.11 | 2.17±0.15 | 0.003* | 0.053 | 0.860 |
| Left Pars opercularis | 2.66±0.12 | 2.58±0.17 | 0.010* | 0.039 | 0.734 |
| Left Pars orbitalis | 2.83±0.24 | 2.77±0.22 | 0.285 | 0.007 | 0.187 |
| Left Pars triangularis | 2.61±0.15 | 2.51±0.19 | 0.011* | 0.038 | 0.722 |
| Left Pericalcarine | 1.57±0.13 | 1.49±0.11 | <0.001** | 0.071 | 0.943 |
| Left Postcentral | 2.03±0.11 | 1.99±0.13 | 0.066 | 0.020 | 0.451 |
| Left Posterior-cingulate | 2.57±0.15 | 2.53±0.16 | 0.247 | 0.008 | 0.212 |
| Left Precentral | 2.40±0.12 | 2.33±0.15 | 0.012* | 0.038 | 0.718 |
| Left Precuneus | 2.34±0.13 | 2.31±0.15 | 0.367 | 0.005 | 0.146 |
| Left Rostral anterior cingulate | 3.02±0.27 | 2.91±0.25 | 0.046* | 0.024 | 0.516 |
| Left Rostral middle frontal | 2.55±0.12 | 2.46±0.16 | 0.004* | 0.050 | 0.833 |
| Left Superior frontal | 2.81±0.14 | 2.72±0.17 | 0.015* | 0.035 | 0.690 |
| Left Superior parietal | 2.18±0.12 | 2.16±0.15 | 0.467 | 0.003 | 0.112 |
| Left Superior Temporal | 2.81±0.16 | 2.73±0.19 | 0.017* | 0.034 | 0.671 |
| Left Supramarginal | 2.61±0.15 | 2.53±0.17 | 0.012* | 0.038 | 0.719 |
| Left Frontal pole | 3.02±0.27 | 2.91±0.30 | 0.095 | 0.017 | 0.386 |
| Left Temporal pole | 3.56±0.30 | 3.50±0.41 | 0.358 | 0.005 | 0.151 |
| Left Transverse temporal | 2.31±0.22 | 2.26±0.23 | 0.202 | 0.010 | 0.247 |
| Left Insula | 3.17±0.17 | 3.07±0.18 | 0.002* | 0.056 | 0.879 |
| Right Caudal anterior-cingulate | 2.62±0.22 | 2.62±0.24 | 0.753 | 0.001 | 0.061 |
| Right Caudal middle frontal | 2.58±0.11 | 2.51±0.16 | 0.024* | 0.030 | 0.617 |
| Right Cuneus | 1.82±0.13 | 1.79±0.13 | 0.127 | 0.014 | 0.332 |
| Right Entorhinal | 3.32±0.41 | 3.34±0.39 | 0.891 | 0.000 | 0.052 |
| Right Fusiform | 2.69±0.15 | 2.60±0.15 | 0.001* | 0.059 | 0.896 |
| Right Inferior parietal | 2.52±0.13 | 2.50±0.16 | 0.658 | 0.001 | 0.072 |
| Right Inferior temporal | 2.94±0.14 | 2.90±0.16 | 0.275 | 0.007 | 0.193 |
| Right Isthmus– cingulate | 2.52±0.25 | 2.40±0.21 | 0.008* | 0.041 | 0.759 |
| Right Lateral occipital | 2.25±0.13 | 2.25±0.13 | 0.909 | 0.000 | 0.051 |
| Right Lateral orbitofrontal | 2.84±0.19 | 2.72±0.17 | 0.001** | 0.069 | 0.937 |
| Right Lingual | 2.02±0.14 | 1.94±0.12 | 0.002 | 0.059 | 0.894 |
| Right Medial orbital frontal | 2.70±0.26 | 2.56±0.18 | <0.001** | 0.073 | 0.948 |
| Right Middle temporal | 2.99±0.16 | 2.93±0.19 | 0.112 | 0.015 | 0.355 |
| Right Parahippocampal | 2.64±0.30 | 2.54±0.29 | 0.074 | 0.019 | 0.433 |
| Right Paracentral | 2.27±0.12 | 2.19±0.15 | 0.007* | 0.044 | 0.782 |
| Right Pars opercularis | 2.70±0.12 | 2.60±0.17 | 0.004* | 0.049 | 0.826 |
| Right Pars orbitalis | 2.93±0.20 | 2.84±0.24 | 0.049* | 0.023 | 0.503 |
| Right Pars triangularis | 2.66±0.17 | 2.55±0.18 | 0.003* | 0.053 | 0.860 |
| Right Pericalcarine | 1.62±0.14 | 1.52±0.11 | <0.001** | 0.132 | 0.999 |
| Right Postcentral | 2.04±0.09 | 1.98±0.12 | 0.009* | 0.041 | 0.752 |
| Right Posterior Cingulate | 2.52±0.14 | 2.50±0.15 | 0.686 | 0.001 | 0.069 |
| Right Precentral | 2.40±0.12 | 2.32±0.15 | 0.008* | 0.042 | 0.760 |
| Right Precuneus | 2.36±0.12 | 2.30±0.15 | 0.028* | 0.029 | 0.598 |
| Right Rostral anterior cingulate | 3.01±0.26 | 2.98±0.22 | 0.628 | 0.001 | 0.077 |
| Right Rostral middle frontal | 2.55±0.13 | 2.49±0.17 | 0.055 | 0.022 | 0.485 |
| Right Superior frontal | 2.81±0.15 | 2.73±0.18 | 0.029* | 0.028 | 0.588 |
| Right Superior parietal | 2.16±0.13 | 2.14±0.14 | 0.450 | 0.003 | 0.117 |
| Right Superior Temporal | 2.84±0.15 | 2.77±0.18 | 0.031* | 0.028 | 0.582 |
| Right Supramarginal | 2.59±0.15 | 2.54±0.15 | 0.083 | 0.018 | 0.412 |
| Right Frontal pole | 3.04±0.30 | 2.88±0.33 | 0.031* | 0.028 | 0.582 |
| Right Temporal pole | 3.58±0.49 | 3.61±0.41 | 0.779 | 0.000 | 0.059 |
| Right Transverse temporal | 2.36±0.17 | 2.32±0.23 | 0.308 | 0.006 | 0.174 |
| Right Insula | 3.16±0.20 | 3.03±0.19 | <0.001** | 0.071 | 0.943 |

*p<0.05; **p<0.00075 (Bonferroni correction)
